# An integrated learning algorithm for early prediction of melon harvest

**DOI:** 10.1038/s41598-022-20799-z

**Published:** 2022-10-28

**Authors:** Chunyang Qian, Taihang Du, Shuguang Sun, Wei Liu, Haiguang Zheng, Jianchun Wang

**Affiliations:** 1 School of Artificial Intelligence, Hebei University of Technology, Tianjin, 300130 China; 2 Institute of Information, Tianjin Academy of Agricultural Sciences, Tianjin, 300192 China; 3 Agricultural Information Center of Zhangjiakou City, Hebei, 075061 China

**Keywords:** Plant sciences, Computational science

## Abstract

Different modeling techniques must be applied to manage production and statistical estimation to predict the expected harvest. By calculating advanced production methods and the rational valuation of different factors, we can accurately capture the variety of growth characteristics and the expected yield. This paper obtained 32 feature variables related to melons, including phenological features, shape features, and color features. The Gradient Boosted Decision Tree (GBDT) network and the Grid Search (GS) hyperparameter seeking method was applied to calculate the degree of importance of all melon fruits' characteristics and construct prediction models for three expected harvest indexes of melon yield, sugar content, and endocarp hardness. To facilitate growers to carry out prediction and estimation in the field without destroying the melon fruits. The reduced feature variables were selected as inputs. The GBDT model was used to provide a significant advantage in prediction compared to both Random Forest (RF) and Support Vector Regression (SVR) methods. In addition, to verify the feasibility of using only reduced feature variables as input for the evaluation work, this study also compares the predictive effects of the model when all feature variables and only reduced feature variables are used. The GBDT prediction model proposed in this paper predicted melon yield, sugar content, and hardness using reduced features as input, and the model R2 could reach more than 90%. Therefore, this method can effectively help growers carry out early non-destructive inspection and growth prediction of melons in the field.

## Introduction

China has become the world's top producer and consumer of melons. 48.7% of the world's melon production in 2020, and per capita consumption is 2.6 times higher than the world average^1^. Beijing, Tianjin, and Hebei are well-known melon-producing areas in China, where melons are crisp, watery, sweet, and well-loved by people. The temperature difference between morning and evening in the north is significant, which helps melons accumulate sugar^2^. The three places' climate conditions and production facilities have their characteristics, and there is a broad space for collaboration in product supply and marketing, technical cooperation, and other aspects.

While breeding new varieties, traits such as growth, flowering, fruiting, fruit size, and color are related to the selection of superior individuals^3^. They are closely related to yield and quality. During melon growth, quality depends not only on its shape and appearance characteristics but also on its soluble solids content, hardness, total acidity, and ripeness, among other factors^4^. In the melon harvesting process, the fruit's weight and maturity are considered the comprehensive evaluation index in the sales process, and the detection of the maturity can protect consumers' interests and improve the processing quality and production-grade of melon to increase market competitiveness^5^. There is a strong demand in the agricultural field to predict its yield and quality^6^. Correlation analysis and prediction of features affecting melon yield and quality can facilitate early decision-making by farmers or plant breeders or enable producers to predict financing aspects before crop harvest^7^.

Machine learning, a branch of AI (Artificial Intelligence), is a practical approach to providing better yield and quality predictions based on multiple features^8^. It can automatically process the relationship between input and output variables and mine the implicit patterns from example samples to "learn" the structural description of these data^9^. Machine learning algorithms can automatically solve significant nonlinear problems and support better decision-making and operations in real-world scenarios without human intervention^10^. Gradient Boosted Decision Tree, as a mature integrated learning algorithm, focuses on effectively reducing the deviation of predicted values from the actual ones. It connects multiple regression tree models in series to form a strong learner, and its base-learner regression tree model has the characteristics of high efficiency and insignificant missing values. The performance is improved by continuously fitting the residuals of the previous tree^11^, which focuses more on the accuracy of the learning model^12^. It also has the advantages of high efficiency, accurate prediction, insensitivity to the original data, and high interpretability of the model^13,14^. Gradient Boosted Decision Tree models have been used to predict stress, soil water content, and remote sensing images in agriculture. Zhang et al. used the GBDT model to build a two-stage identification model for 14 features in high-resolution images of early stress in maize, which can be well applied not only for moisture stress detection in non-destructive measurements but also in the field^15^. Liu et al. established a GBDT model for Soil Moisture (SM) inversion by satellite, and the study showed the excellent performance of GBDT in Soil Moisture Active Passive (SMAP) SM downscaling^16^. Wu et al. used a GBDT model for simulating monthly mean daily ET0 (evapotranspiration) using only local or cross-site temperature data. They showed higher estimation accuracy than other models in local applications^17^. Yang et al. applied GEOBIA (Geographic Object-Based Image Analysis) to crop remote sensing. GBDT and RF have higher classification accuracy and less computation time than SVM and thus are considered more suitable for crop classification that requires a large number of image features^18^.

In this paper, data on fruit phenological features, shape features (including appearance and internal features), and color features (including exocarp and endocarp color features) were recorded during the growth of melons. The GBDT algorithm was used to analyze and predict the importance of features affecting melon fruits' yield, sugar content, and hardness values. The prediction performance and results of two classical regression models, Support Vector Regression (SVR) and Random Forest (RF), are comprehensively compared. Also, the prediction results of the model when all feature variables and only reduced feature variables are compared so that the model can be better applied to non-destructive testing tasks in the field.

## Materials and methods

### Culture implementation and maintenance

The study was conducted at the experimental base of Tianjin Academy of Agricultural Sciences. It was located in Beichen District, Tianjin City, on the east coast of Asia and Europe at mid-latitude, N: 39°18′53.32″ E: 117°13′23.73″. The experiment was carried out in the summer and autumn in the Tianjin area, with temperatures ranging from 16 to 25 °C from August to October, average precipitation of 214 mm, and 59% sunshine percentage in the area. Seedlings were planted on August 5 and harvested on October 16.

The test crop was the thick-skinned melon variety "232", provided by the Institute of Vegetables, Tianjin Academy of Agricultural Sciences. The variety ripened about 35 d after flowering and about 15 d after the melon expansion period. Melon seedlings were transplanted when they had two true leaves and one terminal bud at a planting density of 33,945 plants/ha. Organic fertilizer and high potassium compound fertilizer were applied sufficiently before planting, mixing, and rototilling.

We confirm that the plants used in the article were sampled and measured during field production and comply with international, national and institutional guidelines for this study.

### Measurement methods and instruments

All indicators were evaluated at harvest. Electronic scales were used to weigh weight indicators, vernier calipers to measure shape indicators, hardness meters to measure flesh hardness indicators, sugar meters to measure sugar content, and color indicators were measured using a colorimeter for indicator measurement, and phenological information was recorded manually.

**Figure 1:**
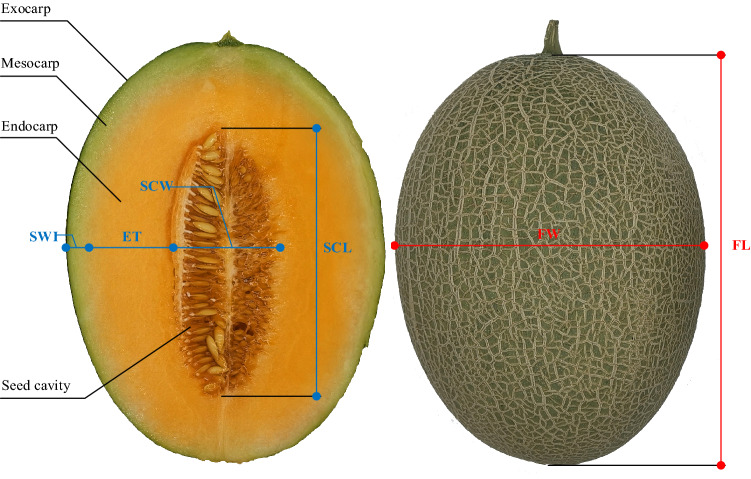
Melon fruit shape features.

The measuring instruments and specifications used in this study mainly include Small-range precision balance (ZG-TP203), specification 5 kg/0.01 g, Shanghai Ranhao Electronics Co., Ltd; Vernier caliper (range: 0 ~ 150 mm, accuracy: ± 0.02 mm), Shanghai Shouxi Work Tools Co., Ltd; 3 NH (NR110 type, measuring aperture: 8 mm) automatic portable colorimeter, Shenzhen San En Shi (3 NH) Technology Co. GY-4 digital display fruit hardness tester, Beijing Jinkolida Electronic Technology Co., Ltd; PAL-1 fruit digital display sugar tester, Japan ATAGO Co. as shown in Fig. 1.

Each complete sample was collected three times for weight index to take the average value. Sugar content value was obtained at the edge of the seed cavity and endocarp, and each half of the sample was collected three times to take the tie-breaker value. Hardness values were collected at the center of the endocarp, and each half of the sample was collected simultaneously on the left and right sides to take the average value. The melon fruit develops from the receptacle and the ovary. The fruit can be divided into the pericarp and the seed cavity. The pericarp is composed of the exocarp, mesocarp, and endocarp, and the mesocarp and endocarp are not clearly defined. Both consist of large, thin-walled cells rich in water and soluble sugars. The shape indexes were measured by vernier calipers for the corresponding position indexes. The description of each index is shown in Table 1.

**Table 1:** Feature variable related description.

Number	Feature name	Measurement meaning	Abbreviation
**Phenological features**			
1	Days of flowering	Affects fruit ripening and time to market	DF
2	Number of days to fruit ripening		NF
	**Appearance features**		
3	Fruit width	Influence the appearance of commerciality and fruit weight^19^	FW
4	Fruit length		FL
5	Fruit shape index	Impact on the appearance of commodity classification^20^	FSI
	**Internal features**		
6	Sum of exocarp and mesocarp fruit widths	Inedible parts	SWI
7	Endocarp thickness	Edible parts	ET
8	Total peel thickness	Total flesh thickness, excluding the seed cavity	TPT
9	Seed cavity width	Size of seed cavity affects weight per fruit; edible portion	SCW
10	Seed cavity length		SCL
**Exocarp color features**			
11 ~ 15	L*a*b*c*h* color space of exocarp	Influence on the determination of fruit ripeness^3,21^	XC_L*, XC_a*, XC_b*, XC_c*, XC_h*
16 ~ 18	Hunter L*a*b* color space of exocarp		XC_HL*, XC_Ha*, XC_Hb*
19 ~ 21	XYZ color space of exocarp		XC_X, XC_Y, XC_Z
**Endocarp color features**			
22 ~ 26	L*a*b*c*h* color space of Endocarp	Influence on the determination of fruit ripeness	NC_L*, NC_a*, NC_b*, NC_c*, NC_h*
27 ~ 29	Hunter L*a*b* color space of Endocarp		NC_HL*, NC_Ha*, NC_Hb*
30 ~ 32	XYZ color space of Endocarp		NC_X, NC_Y, NC_Z

### Gradient boosting decision tree model

Gradient Boosted Decision Tree, an integrated learning algorithm with CART Classification and regression tree (CART) as the base model, is composed of two parts: Decision Tree and Gradient Boosting. Its primary learner is the regression tree, which minimizes the mean square error by constructing a function to fit the elements in the data set. Multiple regression tree models are composed using a combination strategy to obtain the GBDT integrated learner.

A GBDT learning model was developed by combining phenological features, shape features, and color features with melon fruit yield, sugar content, and hardness.

where, ${X}_{i}, {Y}_{i}$ are the corresponding criterion parameters; ${S}_{i}$ is the corresponding leaf node in the regression tree model. $D=\{({x}_{1},{y}_{1}),({x}_{2},{y}_{2}),...,({x}_{m},{y}_{m})\}$ is a dataset containing *m* training samples, each described by *d* feature attributes, i.e.: ${x}_{j}=[{x}_{j1},{x}_{j2},...,{x}_{jd}]$; ${y}_{j}$ is the yield, sugar content, and hardness of the corresponding sample. The basic algorithmic flow of the established GBDT model is as follows^22^.

1. Initialize the base learner ${f}_{0}(x)$:
  1 $${f}_{0}\left(x\right)=\mathit{arg}\underset{a}{min}{\sum }_{j=1}^{m}L\left({y}_{i},a\right)$$
  In which: $L({y}_{i},a)$—loss function; $\mathit{arg}\underset{a}{min}$—the function that determines the value of $a$ taken at the minimum of the value of the loss function.
2. A series of CART regression trees are built, on which the residuals are fitted using a gradient boosting technique. In the $k$ th (*k* = 1,2,…*K*) iteration, for each sample $({x}_{j},{y}_{j})$, GBDT specifies the negative gradient of the loss value as the residual estimate. In this paper, we choose Least mean squared error, as the loss function.
  2 $$L({y}_{i},f({x}_{j}))=({y}_{i}-f\left({x}_{j}\right){)}^{2}$$
  The residual estimates are:
  3 $${R}_{jk}=-\frac{\partial L({y}_{i},f({x}_{j}))}{\partial f({x}_{j})}={y}_{i}-f({x}_{j})$$
3. After determining the residual estimates, the CART regression tree is fitted to obtain the leaf node region of the $k$ th tree as ${c}_{jk}(j=1, 2, \dots J )$, and $J$ is the number of leaf nodes in the regression tree. For each leaf node region, determine the best-fit value ${\beta }_{jk}$ that minimizes the corresponding loss function.
  4 $${\beta }_{jk}=\mathit{arg}\underset{\beta }{min}{\sum }_{{x}_{j}\in {C}_{jk}}L({y}_{j},{f}_{k-1}({x}_{j})+\beta )$$
  5 $${\beta }_{jk}=\mathit{arg}\underset{\beta }{min}{{\sum }_{{x}_{j}\in {C}_{jk}}({y}_{j}-{f}_{k-1}({x}_{j})-\beta )}^{2}$$
4. Update Learner $${f}_{k}({\varvec{x}})$$:
  6 $${f}_{k}(x)={f}_{k-1}(x)+{\sum }_{j=1}^{J}{\beta }_{jk}\cdot \eta$$
  in which: $\eta$—learning rate.
5. After the end of the iteration, a GBDT strong learner is formed, which can be expressed as:
  7 $$F(x)={f}_{0}(x)+{\sum }_{k=1}^{K}{\sum }_{j=1}^{J}{\beta }_{jk}\cdot \eta$$
6. The principle of feature importance calculation can be expressed in the following way^13^: in the description of the approximate $F(x)$, the relative impact of individual input ${x}_{j}$ on the change in $F(x)$ over the distribution of joint input variables ${I}_{j}$ can be expressed as:
  8 $${I}_{j}={\left({E}_{x}{\left[\frac{\partial \stackrel{\wedge }{F(x)}}{\partial {x}_{j}}\right]}^{2}\cdot {\mathit{var}}_{x}\left[{x}_{j}\right]\right)}^{1/2}$$

The global importance of a feature is measured by the average of the importance of the feature in a single tree:

9 $$\stackrel{\wedge }{{I}_{j}^{2}}(T)=\frac{1}{M}{\sum }_{m=1}^{M}\stackrel{\wedge }{{I}_{j}^{2}}({T}_{m})$$

In which: $M$—the number of trees.

The importance of features in a single tree is as follows:

10 $$\stackrel{\wedge }{{I}_{j}^{2}}(T)={\sum }_{t=1}^{L-1}\stackrel{\wedge }{{i}_{t}^{2}}1({v}_{t}=j)$$

In which: $L$—the number of leaf nodes of the tree, $L-1$ is the number of non-leaf nodes of the tree (the constructed trees are binary trees with left and right leaves); ${v}_{t}$—the feature associated with node $t$; ${i}_{t}^{2}$—the reduced value of the squared loss after node splitting.

## Experimental results and analysis

For GBDT, this paper uses GS (grid search method)^23^ to optimize the four hyperparameters on the modeled dataset. Maximum number of iterations is 750, maximum depth is 5, learning rate is 0.05, minimum number of leaves is 1.

### Evaluation metrics

The prediction results of each model were evaluated and compared using the R^2^ (R-squared, goodness-of-fit). Meanwhile, the MAE (Mean absolute error) and RMSE (root mean squared error) metrics were used to comprehensively compare the prediction effects of each model. The TIME (Consumption time) of the model calculation is used to evaluate the efficiency of the model calculation. Among them, MAE reflects the deviation between predicted and actual values, and RMSE reflects the standard deviation of the difference between predicted and actual values.

11 $${R}^{2}=1-\frac{{\sum }_{j=1}^{T}({y}_{j}-{p}_{j})}{{\sum }_{j=1}^{T}({y}_{j}-\stackrel{\_}{y}{)}^{2}}$$

12 $$MAE=\frac{1}{T}{\sum }_{j=1}^{T}|{y}_{j}-{p}_{j}|$$

13 $$RMSE=\sqrt{\frac{1}{T}{{\sum }_{j=1}^{T}({y}_{j}-{p}_{j})}^{2}}$$

In which: $\stackrel{\_}{y}$—the average of the target; ${y}_{j}$—the fruit weight, sugar content and hardness values as noted in the field; ${p}_{j}$—the predicted value of the $j$ th sample estimated by the model.

### Importance analysis of feature variables

**Figure 2:**
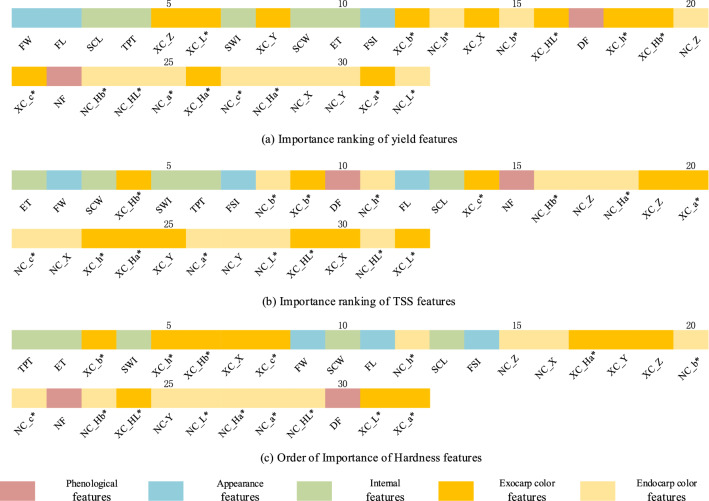
Importance order of feature variables related to yield, sugar content, and hardness.

The importance of each feature variable in the prediction process of melon yield, sugar content, and hardness value was calculated according to the principle of feature importance, as shown in Fig. 2.

Importance analysis was performed according to the importance of the three target feature variables and categories. For yield: Fruit width (FW), Fruit length (FL), Seed cavity length (SCL), Total peel thickness (TPT), and internal character parameters related to endocarp and seed cavity were decisive for yield. For sugar content, the parameters Endocarp thickness (ET), Seed cavity width (SCW), Sum of exocarp and mesocarp fruit widths (SWI), and Total peel thickness (TPT), which are related to the size of the edible part of the fruit, are closely related. For hardness, the internal characteristics Total peel thickness (TPT), Endocarp thickness (ET), Sum of exocarp and mesocarp fruit widths (SWI), and exocarp color features have a significant influence on the hardness index. Phenological characteristics have a more critical effect on sugar content than yield and hardness. Among the color indicators, XC_Hb* and XC_b*, which were spatially related to the yellow-blue color characteristic of the exocarp, had significant associations for both sugar content and hardness, and the exocarp color was more important for all three indicators compared to the endocarp color.

**Figure 3:**
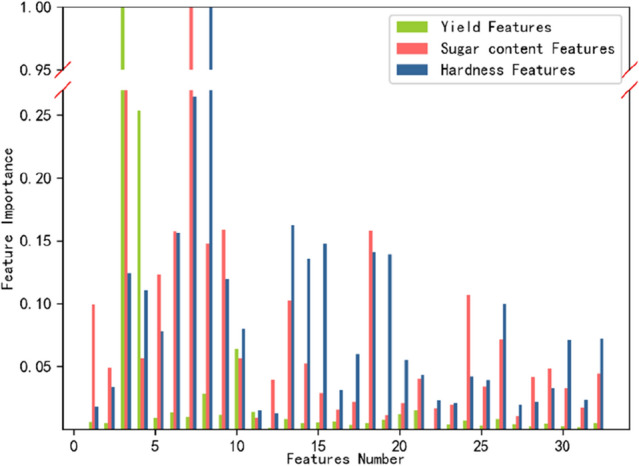
Importance of yield, sugar content, and hardness feature variables.

According to Fig. 3. The essential characteristics for yield, sugar content, and hardness were Fruit width (FW), Endocarp thickness (ET), and Total peel thickness (TPT). Fruit width (FW) had more influence on yield and sugar content than other features, and ET affected both sugar content and hardness. The influencing factors of yield index were mainly concentrated on appearance features, and the influencing factors of sugar content and hardness index were mainly concentrated on internal features. At the same time, the color features of outer peel also had a relatively significant influence on them.

### Predictive effect analysis

**Figure 4:**
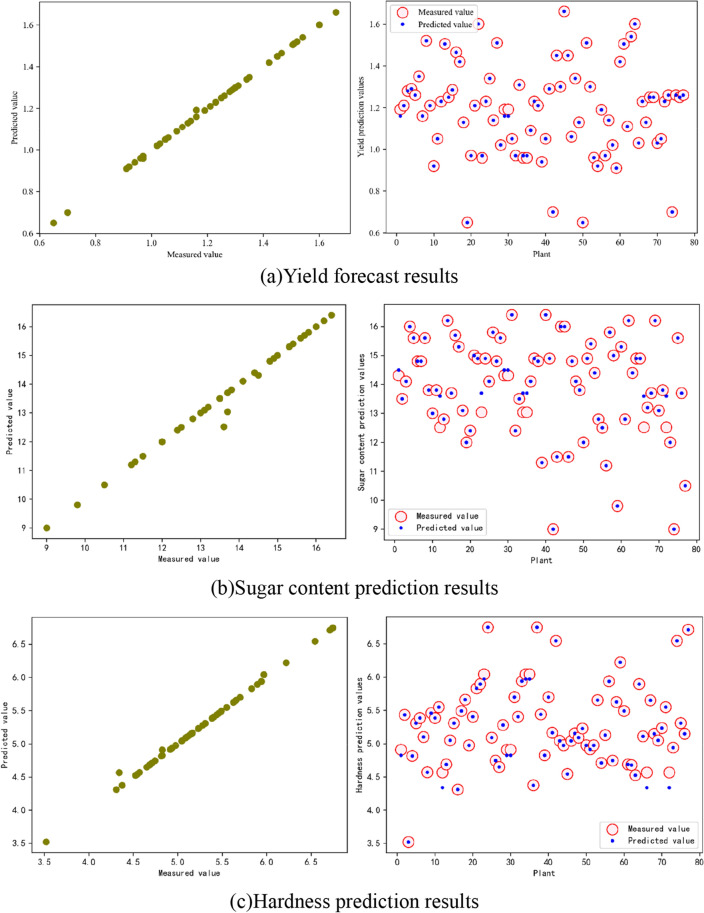
GBDT prediction effectiveness.

The GBDT learning model developed in this paper predicts melon yield, sugar content, and hardness values for 32 feature variables, including phenological features, appearance features, internal features, exocarp color features, and endocarp color features, respectively. With the 255 samples obtained, the learning model randomly divides the training and testing sets in a 7:3 ratio by setting a random seed number in the training and testing phases. The evaluation metrics are shown in Table 2. The prediction results are shown in Fig. 4.

**Table 2:** Predictive metrics.

	RMSE	MAE	R^2^	TIME
Yield forecast	0.041421	0.001716	0.999048	1.0118
Sugar content prediction	0.274511	0.075356	0.976626	0.909321
Hardness prediction	0.014827	0.121767	0.993375	0.853631

## Discussion

In order to facilitate growers to be able to operate in the field and help them to estimate them in the early stages of planting, a reduced feature prediction model with melon phenological features, appearance features, and exocarp color features as inputs were constructed and discussed. The purpose is used to compare the feasibility of using prediction models for melon yield, sugar content, and hardness under non-destructive conditions.

**Figure 5:**
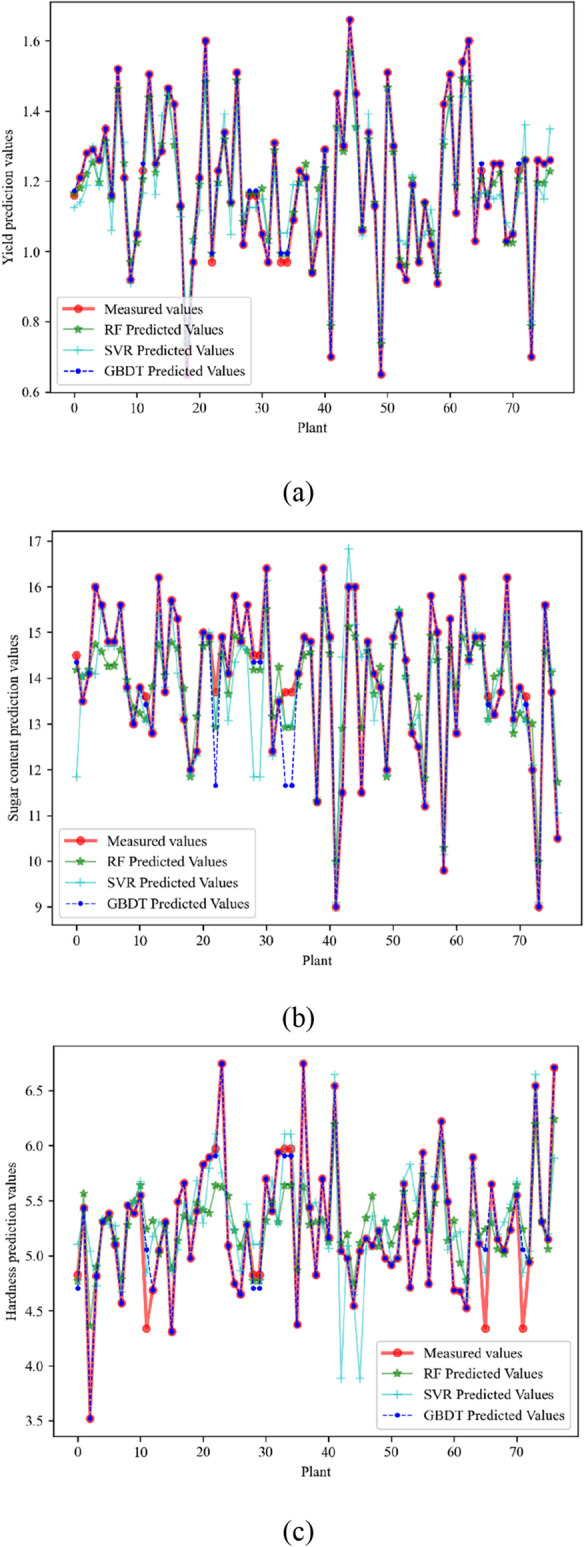
Comparison of yield (**a**), sugar content (**b**), and hardness (**c**) prediction results of different models with the reduced features as inputs.

At the same time, to evaluate and test the prediction effects of GBDT learning models on melon yield, sugar content, and hardness, this paper establishes GBDT, SVR, and RF simultaneously and with constant input and output feature parameters and sample data set division. The comprehensive analysis to compare the prediction results is shown in Fig. 5, and the evaluation indexes are shown in Table 3. R2, RMSE, MAE, and TIME were used to predict the results of each model. The RF parameters: the number of iterations is 750, the maximum depth is 5, and the minimum number of leaf nodes is 1. The SVM parameters: the kernel uses 'RBF', C is 100, and the learning rate is 0.001.

**Table 3:** Predictive metrics with the reduced features as inputs.

Predicted target	Models	RMSE	MAE	R^2^	TIME
Yield forecast	GBDT	0.047709	0.002276	0.999001	0.328718
	RF	0.197566	0.039032	0.941989	0.912089
	SVR	0.247726	0.061368	0.899188	0.001988
Sugar content prediction	GBDT	0.303210	0.091936	0.939922	0.326918
	RF	0.818521	0.669977	0.764265	0.920649
	SVR	0.930617	0.866048	0.47289	0.003874
Hardness prediction	GBDT	0.187256	0.035065	0.944255	0.328509
	RF	0.550309	0.302840	0.568469	0.922128
	SVR	0.563522	0.317557	0.455216	0.004019

The comparison shows that since the growth process of decision trees is to select and partition features continuously, GBDT, which consists of a large number of decision trees, has the inherent advantage that the importance ranking of features can be easily obtained and is highly interpretative. The Support Vector Regression (SVR) model is used for regression using the idea of Support Vector Machine (SVM), which allows for an ε distance between the predicted and true values of the model to improve generalization compared to traditional regression models, and has an advantage for nonlinear^24^. Gradient Boosted Decision Tree and Random Forest (RF) final results are determined by multiple trees together, and different from RF is the idea of training the base learner. RF uses the Bagging method, which cannot improve the bias. In contrast, GBDT uses Boosting method, and each iteration is weighted to the sample based on the prediction result of the previous iteration. As iterations continue, low bias can be guaranteed to improve the model's generalization ability.

**Figure 6:**
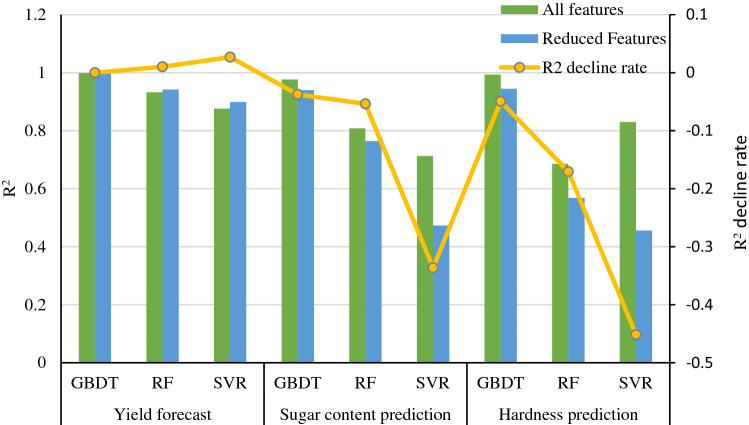
Comparison of R^2^ indicators for different models before and after feature reduction.

As shown in Fig. 6, by comparing the R2 indicators of the three models with all the feature variables and the reduced feature variable as inputs, it can be seen that the GBDT model has a higher R2 for yield prediction than the sugar content and hardness models before and after the feature reduction. In predicting yield using the three different models, the use of reduced features improved the R2 of the model compared to all feature variables as input. On the contrary, reducing features as input decreased the R2 of all three prediction models in predicting sugar content and hardness, and the SVR model predicted a significant decrease in R2 of more than 30%. It related to the importance of all the feature variables in the prediction models discussed earlier, where reduced features were mostly located at the top of the variable importance ranking in the yield prediction models, and FW and FL were close to 100% in the feature importance scores. In contrast, more internal features, such as ET, TPT, and SCW, in the sugar content and hardness prediction models had higher importance scores. Adding some internal fruit measures could further improve the prediction accuracy of sugar content and hardness.

The GBDT prediction model proposed in this paper was used to predict melon yield, sugar content, and hardness using reduced features as input, and the model R2 could reach more than 90%. Therefore, using non-destructive feature datasets to predict melon fruit yield, sugar content, and hardness can achieve high prediction accuracy, and the method is feasible.

## Conclusions

In this paper, we propose a machine learning method using GBDT and GS (Grid Search Method) optimization-seeking hyperparameter method for melon varieties in northern China to analyze the importance of the characteristic variables affecting melon yield, sugar content, and hardness and to successfully predict the test data. A significant benefit of early forecasting is that it allows early assessment and preparation for those involved in breeding new varieties, making production decisions, and seeking financial support. To improve the model's usefulness, combined with hardware devices for use in the field without destroying the fruit itself, this study screened non-destructive features of melon fruit, including melon phenological features, appearance features, and exocarp color features, to construct a non-destructive prediction model. Through the analysis of the study, the model still has acceptable prediction results compared to all features used as input. At the same time, the model training time decreases, and the running efficiency improves due to the reduction of feature variables. Therefore, in the future, by embedding the prediction model into the vision inspection equipment, it will be easy to achieve non-destructive, accurate, and fast prediction and evaluation work in the field.

## Data Availability

The availability of some or all of the data generated or analyzed in this study is restricted and the entire data cannot be made public until the variety is officially licensed as a variety. It is not publicly available, but are available from the corresponding author on reasonable request.
